# Epigenetic Symphony in Diffuse Large B-Cell Lymphoma: Orchestrating the Tumor Microenvironment

**DOI:** 10.3390/biomedicines13040853

**Published:** 2025-04-02

**Authors:** Andreea-Daniela Caloian, Miruna Cristian, Elena Calin, Andreea-Raluca Pricop, Stelian-Ilie Mociu, Liliana Seicaru, Sorin Deacu, Nicolae Ciufu, Andra-Iulia Suceveanu, Adrian-Paul Suceveanu, Laura Mazilu

**Affiliations:** 1 Faculty of Medicine, “Ovidius” University of Constanta, 900470 Constanta, Romania; elena.beca@yahoo.com (E.C.); sorin.deacu@365.univ-ovidius.ro (S.D.); office@ovidius-ch.ro (N.C.); andrasuceveanu76@gmail.com (A.-I.S.); asuceveanu@yahoo.com (A.-P.S.); lauragrigorov@gmail.com (L.M.); 2 Department of Hemato-Oncology, “Ovidius” Clinical Hospital, 900470 Constanta, Romania; mociustelian@gmail.com; 3 Center for Research and Development of the Morphological and Genetic Studies of Malignant Pathology-CEDMOG, “Ovidius” University of Constanta, 900470 Constanta, Romania; 4 Department of Forensic Medicine, “Sf. Apostol Andrei” Emergency County Hospital, 900439 Constanta, Romania; 5 Department of Dermatology, “Sf. Apostol Andrei” Emergency County Hospital, 900591 Constanta, Romania; andreearaluca24@yahoo.com; 6 Department of Clinical Patology, “Sf. Apostol Andrei” Emergency County Hospital, 900591 Constanta, Romania; lilianamcn@gmail.com; 7 Department of Gastroenterology, “Sf. Apostol Andrei” Emergency County Hospital, 900591 Constanta, Romania

**Keywords:** diffuse large B-cell lymphoma, epigenetic, tumor microenvironment

## Abstract

DLBCL is a testament to the complexity of nature. It is characterized by remarkable diversity in its molecular and pathological subtypes and clinical manifestations. Despite the strides made in DLBCL treatment and the introduction of innovative drugs, around one-third of patients face a relapse or develop refractory disease. Recent findings over the past ten years have highlighted the critical interplay between the evolution of DLBCL and various epigenetic mechanisms, including chromatin remodeling, DNA methylation, histone modifications, and the regulatory roles of non-coding RNAs. These epigenetic alterations are integral to the pathways of oncogenesis, tumor progression, and the development of therapeutic resistance. In the past decade, the identification of dysregulated epigenetic mechanisms in lymphomas has paved the way for an exciting field of epigenetic therapies. Crucially, these epigenetic transformations span beyond tumor cells to include the sophisticated network within the tumor microenvironment (TME). While the exploration of epigenetic dysregulation in lymphoma cells is thriving, the mechanisms affecting the functions of immune cells in the TME invite further investigation. This review is dedicated to weaving together the narrative of epigenetic alterations impacting both lymphoma cells with a focus on their infiltrating immune companions.

## 1. Introduction

Diffuse large B-cell lymphoma is the most common NHL subtype, accounting for approximately 30% of NHL and 80% of all aggressive lymphomas. The incidence of DLBCL increases with age and there is a higher male predominance. It is a very heterogeneous disease with different morphologies, genetic, behavior, and aggressive features. The characteristics of DLBCL are represented by diffuse proliferation of medium or large lymphoid B-cells typically expressing CD19, CD20, CD79a, PAX5, and surface or cytoplasmic immunoglobulin. DLBCL is also characterized by the expression of other markers, like BCL2 and BCL6, CD10, and IRF4/MUM1. CD5 expression can be found in approximately 30% of DLBCL, and it is associated with a poorer prognosis [1,2]. Defined by heterogeneity, we find differences in clinical presentation, pathological characteristics, and molecular features [3,4,5,6]. There are three main types of DLBCL, described in the last classification of Hematolymphoid Tumors, WHO-HAEM5, activated B-cell-like DLBCL accounting for 50–60%, germinal centre B-cell-like DLBCL accounting for 40–50%, and unclassified 10–15%. ABC and unclassified subtypes are commonly named non-GCB [1]. This classification is based on gene expression profiling and on the cell of origin, helping in subclassifying DLBCL into different molecular subtypes that further correlate with different prognostic implications. However, even with this classification, we are not able to provide a full picture of the DLBCL complexity, emphasizing the need for deep molecular classification. The ABC-DLBCL subtype mainly consists of large cells derived from peripheral activated B-cells that frequently have immunoblastic characteristics. The gene profile of ABC-DLBCL is a post-CGB profile lacking germinal centre markers and constitutive activation of BCR signaling and the nuclear factor kappa B pathway and the expression of MUM1/IRF4. ABC-DLBCL is associated with a poorer prognosis and poor response to standard treatment. GCB-DLBCL is mainly formed by large cells, derived from normal germinal center B-cells, resembling centroblasts and mimic the differentiation mechanisms occurring in normal germinal centers. This subtype is characterized by the expression of GC markers like BCL6 and CD10 as well as hypermutated immunoglobulin and somatic mutations. GCB DLBCL is associated with a better response to treatment and better overall survival [7,8,9]. All these histological and immunohistochemistry classifications are very useful in understanding the pathogenesis of DLBCL, but do not provide important utility, because they do not consider the multitude of changes that appear within the genome, that cannot be obtained by using classical histological and immunohistochemical techniques. Recent data showed that new molecular subclassifications add more subtypes to DLBCL [6,10]. Chapuy et al. classified DLBCL cells into six subtype cells into C (clusters), C0 if a genetic driver could not be identified, and C1 to C5, depending on the pattern of gene expression with defining genetic drivers. C1 has an alteration in the *NOTCH2* pathway, C2 is associated with *TP53* mutation and the loss of *CDKN2A* and *RB1*, C3 is associated with *BCL2* and chromatin modifier mutations, C4 was characterized by mutations in four linker and four core histone genes, and C5 is associated with 18q gain and increased extranodal involvement [11]. A study published in 2018 by Schmitz et al, who evaluated 574 DLBCL patients using exome and transcriptome sequencing on biopsy samples, identified four genetic DLBCL subtypes: subtype BN2, based on *BCL6* fusions and *NOTCH2* mutations, subtype EZB, based on *EZH2* mutations and *BCL2* translocations, subtype MCD, based on the co-expression of *MYD88*^L265P^ and *CD79B* mutations, and N1 subtype based on the presence of *NOTCH1* mutation. All these subtypes are found to have different phenotypes, different responses to treatment, and different outcomes, with better survival for the BN2 and EZB groups compared with MCD and N1 groups that appear to have poorer outcomes [12]. Another research conducted by Lacy et al. in 2020 [13] classified DLBCL also in five groups *BCL2*, *NOTCH2*, *MYD88*, *SOCS1/SGK1* and *TET2/SGK1*. The *BLC2* group display t(14;18) translocation and other mutations in *BCL2* pathways. The *NOTCH2* subtype include mutations in *NOTCH2*, *BCL10*, and *CD70*, and correspond to C1 in the Chapuy classification. The *MYD88* subgroup is defined by *MYD88* mutation, and it is similar to C5 in the Chapuy classification and MCD in Schmitz’s study. *SOCS1/SGK1* displays mutations that are common to primary mediastinal B-cell lymphoma (PMBL) and appear to represent a subgroup of C4. The *TET2/SGK1* subgroup has characteristic mutations like *KRAS*, *SGK1*, or *TET2*, and appears to be another subgroup of the C4 Chapuy classification [10,13]. Wright et al. developed another classification, named “LymphGen”, that has more clinical usefulness. This classification is based on previous classifications performed by Chapuy and Schmitz and include seven groups classified by features and incidence—A53 presenting aneuploidy and *TP53* inactivation; *BN2* represented by *NOTCH2* signaling with *BCR*-dependent NF-κB immune evasion and a loss of CD70; MCD with *BCR*-dependent NF-κB immune evasion; N1 characterized by altered B-cell differentiation and *NOTCH1* signaling; *ST2* with *JAK/STAT3* signaling with NF-κB activation and EZB with chromatin modification and *PI3* kinase signaling and *S1PR2-GNA13* inactivation. EZB groups were divided into two subgroups of EZB/MYC positive and EZB/MYC negative [14].

Epigenetics refers to the complex modifications that influence gene expression without changing the underlying DNA sequence. This phenomenon is primarily driven by processes like DNA methylation and post-translational modifications of histones, which are crucial for the regulation of transcriptional activity. The way that epigenetic changes interact is important for controlling how genes are expressed in different situations. These changes are essential for gene expression, which is necessary for cells to grow and develop properly. Molecular changes greatly impact gene expression, so they play a key role in how cells differentiate and grow. The epigenome plays a pivotal role in the proper development of lymphocytes, a subset of white blood cells critical to the immune system. These cells orchestrate a robust and coordinated immune response against pathogens [15]. Disruptions in the epigenome are often linked to various types of cancer in humans. These changes can play a key role in cancer development by causing tumor suppressor genes to be turned off. Normally, these genes help stop uncontrolled cell growth. These changes in how genes are expressed can significantly impact the tumour microenvironment, which is the area around the tumor. This can change how the immune system responds to the tumor [16,17]. 

In lymphomas, a group of different blood cancers, these changes in gene expression are very common in many of its subtypes. The recent advancements in gene sequencing technologies, especially next-generation sequencing, have transformed the landscape of lymphoma classification. With the ability to conduct mutational profiling, researchers can gain invaluable insights into the genetic makeup of lymphomas, frequently identifying mutations in the genes associated with epigenetic regulation. Detecting these epigenetic irregularities offers considerable diagnostic promise, as specific distinct mutational profiles can provide crucial information that aids in making particular and targeted diagnoses, ultimately guiding treatment decisions and improving patient outcomes [18].

DLBCL is represented by malignant cells that constantly interact with the TME. This is composed of different cell types interacting with each other to promote immune evasion [19]. This reciprocal interaction between lymphoma cells and immune cells within the TME plays a key role in cancer initiation and progression [20]. Further, immune cell function in the TME is affected by epigenetics, which will provide a favorable environment for tumor growth. In conclusion, the epigenetic influence on both tumor cells and immune cells promotes tumor growth. Cancer cells can escape the immune system, affecting how lymphomas respond to treatment. The complex ways that both tumor and immune cells interact present a valuable chance to disrupt their relationship. By using these methods, we can create new treatment strategies that show great promise in the fight against cancer [21].

## 2. Epigenetic Modifications in DLBCL

Epigenetics, a term first use by Conrad Hal Waddington in 1942, refers to the interactions between the developmental environment and the genome. It is defined as the study of heritable changes in gene function that do not alter the DNA sequence. Epigenetic changes influence the chromatin structure and regulate gene expression. Unlike genetic changes, these modifications are reversible and include DNA methylation, histone modifications, and non-coding RNA [22,23].

Recent advancements have enabled a better understanding of the significance of the epigenetic modifications in both normal hematopoiesis and the oncogenesis of hematologic malignancies [24,25].

### 2.1. DNA Methylation

DNA methylation is a predominant mechanism defined by the process in which DNA methyltransferases transfer a methyl group to the C-5 position of the cytosine ring in DNA. This was the first epigenetic alteration described 30 years ago, and also it is the most studied. DNA methylation can occur in the centromeres, dormant X chromosomes, telomeres, and in repetitive DNA sequences. The role of DNA methylation is important in numerous processes, for example, imprinting and the maintenance of genomic stability. DNA hypo- and hypermethylation of the tumor suppressor gene promoters, represents one of the most important drivers of carcinogenesis and a hallmark of cancer cells [26,27] This epigenetic mechanism occurs primarily in promoter regions, where methylation regulates gene expression by silencing transcription. *BCL6* has an important role in the development of B-cell lymphomas. For example, cytidine deaminase influences the reduction in *BCL6* gene expression by *DNMT1*, resulting in growth inhibition and apoptosis in DLBCL cells [28,29]. Abnormal DNA methylation is found in high levels in DLBCL and correlates with a poorer prognosis. Frazzi et al. show in their research that DLBCL patients have significant DNA methylation in genes like *DAPK1*, *KLF4*, or *SPG20* [30,31]. In DLBCL, the hypermethylation of promoter regions is an important characteristic, as shown by Shawky et al., reporting significantly higher promoter methylation in tumor suppressor genes in DLBCL samples compared to normal samples after the evaluation of 75 DLBCL and 30 normal samples [32]. Follow-up research confirmed an increased promoter methylation in key tumor suppressor genes such as *MYC* and *SLIT2*, likely leading to reduced gene expression and contributing to tumor growth. In contrast, *KIF23* showed that the hypermethylation of its promoter correlated with increased gene expression in DLBCL [33,34]. *KIF23* promoter hypermethylation also has an increased expression in DLBCL and may influence DLBCL development [35]. Another link between DNA methylation and DLBCL is the influence on the progression of the disease; for example, the reduced expression of Cadherin-23 due to DNA methylation has been associated with a poor prognosis in DLBCL patients. CDH23 has an important role in the immune cell infiltration of DLBCL, being implicated in cell cycle, cell adhesion, cancer cell growth, drug catabolic process DNA replication and repair, or leukocyte-mediated immunity [36]. DNA methylation can impact the response to treatment, for example CART19 therapy [37].

The *DNMT* family consists of several members, including *DNMT1*, *DNMT2*, *DNMT3A*, *DNMT3B*, and *DNMT3L*. However, only *DNMT1*, *DNMT3A*, and *DNMT3B* have been implicated in lymphopoiesis and the early stages of B-cell activation [38,39]. *DNMT1* is essential in several lymphomas, and particularly in diffuse large B-cell lymphomas, where it is commonly expressed and promotes cell cycle progression and DNA replication. The *AID-DNMT1* complex inhibits *BCL6* expression, resulting in cell apoptosis and reduced tumor growth in DLBCL xenograft models [40,41].

*DNMT1* and *DNMT3B* are the most frequently overexpressed and mutated *DNMT*s in lymphomas. *DNMT3A* mutations are found in about 11–20% of T-cell lymphoma patients. These mutations typically result in a complete loss of function, which may contribute to the development of these lymphomas [42]. The overexpression of *DNMT3B* is involved in the progression of lymphomas, particularly in Burkitt lymphoma (BL). Approximately 85% of Burkitt lymphoma (BL) patients display *DNMT3B* overexpression, which contributes to DNA methylation in conjunction with *DNMT1* [43,44]. This overexpression results from the direct binding of the oncoprotein *MYC* to the promoter region of *DNMT3B*. Additionally, in DLBCL, *DNMT3B* overexpression has been linked to a poorer prognosis, treatment resistance, and disease progression [45].

Aberrant DNA methylation is a key epigenetic change that contributes to the development and progression of lymphomas; for example, follicular lymphoma and diffuse large B-cell lymphoma show increased levels of this methylation, which worsens with the evolution of the disease and links to poorer outcomes [46]. 

This shows that abnormal methylation can have different effects depending on the gene, indicating its crucial role in DLBCL development and progression. DNA methylation in B-cell malignancies was also linked to disease progression. Targeting the *SP3* promoter, affected by DNA methylation, can promote B-cell lymphomas. 

Interleukin-10 production, essential for regulatory B-cells, is influenced by specific DNA methylation patterns. Studies show that the increased expression of the protooncogene *MYC* correlates with the methylation of its enhancer region, which is associated with genes that drive lymphomagenesis. *DYNLL1* enhances *MYC* function and is implicated in B-cell lymphoma progression. Additionally, the decreased expression of CDH23 due to DNA methylation is linked to a poor prognosis in diffuse large B-cell lymphoma (DLBCL) [35,47,48].

DNA demethylation requires DNA demethylases which include ten-eleven translocation methyl-cytosine dioxygenase (TET). *TETs* are erasers that remove the methyl group from the CpG site. *TET2* mutations can occur in conjunction with the base excision repair machinery, but it also activates transcription independently. *TET2* is a member of the TET family of proteins, and it is a tumor suppressor gene in DLBCL. It is also one of the early mutations that appear during the evolution of DLBCL [25,49,50]. Recent research published in 2024 that evaluated 66 patients with DLBCL concluded that there is a substantial heterogeneity of methylation in DLBCL patients, and DNAm patterns could not be used to distinguish between germinal center B-cell-like and non-GC DLBCL cases. Cases that were treated with chemo-immunotherapy regimens like R-CHOP had high levels of hypomethylation and high levels of CpG island hypomethylation associated with a poorer prognosis and decreased survival [51].

### 2.2. Histones Modifications

Histone modifications include acetylation, citrullination, methylation, phosphorylation, ADP–ribosylation, and ubiquitination. In lymphomas, the most frequent aberrations are the acetylation and methylation of histones [52]. Histone modifications are essential in epigenetic studies. The nucleosome, the basic unit of chromatin, consists of an octamer of histones and 147 DNA base pairs. This octamer contains two copies of each histone: H2A, H2B, H3, and H4. Histones have c-terminal and n-terminal binding regions, and their protruding tails undergo various post-translational modifications [46,53,54]. The enzymes that are involved in histone modifications are classified according to their action as readers, writers, or erasers. The writers add and modify histones, readers can recognize specific regions within nucleosomes, and erasers can remove regulated gene expressions [55].

Histone acetylation is a key modification that affects the gene expression in B-cell non-Hodgkin lymphomas through transcriptional regulation. Histone acetyltransferases (HATs) add acetyl groups to histones, while histone deacetylases (HDACs) remove them, impacting gene expression. Eighteen HDACs have been identified and classified into four groups. Class I includes HDAC1, HDAC2, HDAC3, and HDAC8. Class II is divided into class IIa, including HDAC4, HDAC5, HDAC7, and HDAC9, and class IIb which includes HDAC6 and HDAC10. Class IV has only HDAC11. Classes I, II, and IV are metal dependent, using zinc ions, whereas class III are NAD+ dependent and use NAD+ for catalysis [56].

DLBCL features a high expression of HAT1, HDAC1, and HDAC2. Elevated HDAC2 levels are associated with shorter survival, suggesting it may serve as a poor prognostic marker [57]. Conversely, a higher HDAC6 expression is linked to better outcomes in DLBCL [58]. Histone methylation occurs on lysine (K) and arginine (R) residues at the N-terminal ends of histones H3 and H4, primarily driven by histone methyltransferases (HMTs). This process is crucial in lymphomas. For instance, PRMT5, an arginine methyltransferase, is overexpressed in mantle cell lymphoma (MCL) and diffuse large B-cell lymphoma (DLBCL), underscoring the relevance of histone methylation in these diseases. The Epstein–Barr virus (EBV) also impacts histone modification through lysine-specific demethylase 2B (KDM2B). Additionally, lysine-specific demethylase 1 (LSD1) plays a vital role by demethylating H3K4me2, which represses tumor suppressor genes. This knowledge has led to the development of LSD1 inhibitors, such as ZY0511, which exhibit antitumor effects in DLBCL [59,60]. The enhancer of zeste homolog 2 (*EZH2*) represents an important HMT in DLBCL. Its action is to modulate the trimethylation of histone H3 position 27 (H3K27) during the differentiation and proliferation of cells, with the result of abnormal cell proliferation and lymphomagenesis. In DLBCL, *EZH2* somatic heterozygous mutations are found in up to 30% of cases. *EZH2* activity is increased, targeting its promoter, by HMGA1, a high-affinity protein. *EZH2* increased activity is related to a somatic mutation that replaces the amino acid, tyrosine 641 (Y641) GCB-DLBCL [61].

The P300/CBP family are epigenetic writers with a very important activity in normal hematopoiesis and from this family, two members have an important role in DLBCL—as CREB-binding protein (CREBBP) also known as CBP or KAT2A and EP300 also named P300 or KAT2B. These enzymes are HATs, because they acetylate histone H3 and regulate gene expression. Both *CREBBP* and *EP300* are frequently found in DLBCL. *CREBBP* mutations are found in around 30% of DLBCL patients and are associated with a poorer prognosis in these patients [12]. *EP300* is found to be mutated in up to 15% of cases and appear to be mutually exclusive with *CREBBP* mutation [11,12]. *EP300* mutation appears to increase M2 macrophage infiltration, and it is associated with increased levels of IL-10, which are immunosuppressive [62].

### 2.3. MicroRNAs (miRNAs)

Non-coding RNAs are transcripts that do not encode proteins and include microRNA (miRNA), long non-coding RNA (lncRNA), and circular RNA (circRNA). The role of non-coding RNAs is currently increasing [63]. The latest classification of non-codingRNAs include two major classes, regulatory non-codingRNAs and housekeeping non-codingRNAs. The regulatory class is further subdivided into two short-chain classes, which include long non-coding RNAs (lncRNAs), miRNAs, piwi-interacting RNAs (piRNAs), and small inhibitory RNAs (siRNAs) [63,64]. MicroRNAs (miRNAs) are intensively studied. These are short, about 22 nucleotides long, and regulate gene expression after transcription [65,66]. In diffuse large B-cell lymphoma (DLBCL), many miRNAs, such as miRNA-155 and miRNA-21, are deregulated [67]. For example, the overexpression of miR-130b alters Th17 cells, and miR-155 worsens tumor progression by affecting the PD-1/PD-L1 pathway [68]. 

Recent studies reveal significant miRNA targets in B-NHL related to tumor growth. A specific miRNA signature can differentiate between germinal center B-cell-like DLBCL (GCB-DLBCL) and activated B-cell-like DLBCL (ABC-DLBCL), notably including miRNAs 155, 21, and 221 [65]. The role of miRNA-155 in ABC-DLBCL has been supported by independent research. Additionally, a unique miRNA signature has been identified to distinguish Burkitt lymphoma (BL) from DLBCL [69,70]. In terms of lncRNA, 2632 novel lncRNAs are differentially expressed in DLBCL compared to control B-cells, and 17 lncRNAs can differentiate between GCB and ABC-DLBCL [71,72]. Notably, the lncRNA NKILA is often hypermethylated in DLBCL, and its silencing leads to increased cell proliferation and reduced cell death through NF-kB signaling. Research on circRNA expression in lymphomas is limited [48,73,74]. Dahl et al. created a circRNA expression map for various B-cell malignancies, which distinguishes between different types [75]. Hu et al. found several differentially expressed circRNAs in DLBCL tissues. Specifically, circ-APC (hsa_circ_0127621) is downregulated in DLBCL tissues, cell lines, and plasma, impairing DLBCL cell proliferation and tumor growth in laboratory studies [76].

LncRNAs can be found in the cytoplasm and nucleus and are composed of more than 200 nucleotides [64]. Recent data are suggesting that lncRNAs are implicated in the regulation of TME cells impacting the immune response [77]. One research that evaluated lnc-RNAS in DLBCL patients evaluated 116 samples via RNA sequencing data, and the result showed 2.632 novel, multi-exonic lncRNAs, with two-thirds that are not expressed in normal B-cells and more than one-third with different expressions between GCB-DLBCL and ABC-DLBCL, but further studies are needed to identify the potential role in tumor genesis [77].

CircRNAs are novel lncRNAs and appear to be ubiquitously expressed in mammalian cells [78]. Data suggest that circRNAs have a role in the resistance to immunotherapy, modifying TME and impacting the response to treatment [79]. In DLBCL patients, circPCBP2 appear to be increased alongside with decreased levels of miR-33a/b, and this can be associated with a decreased response to chemotherapy. On the other side, decreased levels of circPCBP2 associated with the overexpression of miR-33a/b appear to increase tumor cell apoptosis and increase the response to chemotherapy [80].

### 2.4. Chromatin Remodeling

Chromatin remodeling is essential in tumor development as it regulates gene expression. The SWI/SNF complex, a key member of chromatin regulators, is mutated in over 20% of tumors [81,82]. In lymphomas, changes in chromatin remodeling complexes, especially the SWI/SNF complex, are common. The ARID1A gene often has mutations, with a 17% mutation rate found in a study of 29 patients with Burkitt lymphoma. These mutations cause important changes in the chromatin structure and function, which can affect how tumors start, grow, and respond to treatment. Thus, studying these mutations in B-NHLs is vital for understanding the disease’s pathophysiology [83]. Representative epigenetic regulators and their role in DLBCL are shown in Table 1. 

## 3. Components of the Tumor Microenvironment 

The tumor microenvironment (TME) is a complex network of cellular and non-cellular components that surround and interact with tumors. It plays a vital role in tumor development and progression. The TME includes an immune microenvironment with dendritic cells (DCs), cells like B and T lymphocytes, natural killer (NK) cells, tumor-associated macrophages (TAMs), myeloid-derived suppressor cells (MDSCs), and neutrophils. It also contains a non-immune microenvironment consisting of cancer-associated fibroblasts (CAFs), an extracellular matrix (ECM), mesenchymal stromal cells, and various secreted molecules such as chemokines and cytokines [85,86,87,88,89]. The TME varies by cancer type and can predict the patient outcomes, particularly in lymphomas. For example, it makes up about 50% of indolent B-cell lymphomas but is lower in aggressive types like DLBCL [90]. In DLBCL, the TME contains diverse cell types with different functions. Flow cytometry and transcriptional analysis can reveal the proportions and states of these cells. Recent methods using artificial intelligence have identified distinct “lymphoma microenvironments” within DLBCL, each with unique characteristics. These include the depleted LME germinal center-like LME, the inflamed and immunosuppressive LME, and the mesenchymal LME [91,92].

### 3.1. Lymphoid Cells

#### 3.1.1. B-Cells

B-cell receptors are anchored or surface immunoglobulins. B-cell receptors undergo somatic hypermutation and switches in the germinal center. The differentiation of plasma cells and plasmablasts is the result of the activation of B-cells and the secretion of antibodies. In DLBCL, the presence of naïve B-cells and plasma cells plays a significant role in prognosis. A study that included DLBCL patients found that a higher percentage of normal B-cells was linked to better survival rates and more favorable clinical outcomes [91,92]. However, a separate analysis of 539 DLBCL samples using CIBERSORT revealed that B-cells and plasma cells did not significantly correlate with survival rates [93].

#### 3.1.2. T-Cells

Lymphocytes, a type of white blood cell, originate from lymphoid progenitor cells and include B-cells, T-cells, and NK cells. These lymphocytes are present in the tumor microenvironment (TME) of lymphomas. T-cells are vital for immune surveillance and disease progression, leading to extensive research on them in various malignancies, including lymphomas [94]. The T-cell compartment has several subpopulations: CD4+ T-helper cells (Th), CD4+/FOXP3+ regulatory T-cells, and CD8+ cytotoxic T-cells (CTLs). Th-cells include Th1, which produce IL-2, IFN-γ, and TNF-β to activate CTLs and other immune cells, and Th2, which release IL-4, IL-5, IL-6, and IL-10, encouraging tumor growth through CD40-CD40L interactions [95]. In diffuse large B-cell lymphoma, the growth of certain genes, IL-10RA and IL-10RB, helps the tumor survive. Lymphoma cells can produce a protein called PD-L1. At the same time, T-cells (both CD4+ and CD8+) have a receptor called PD-1. When these two interact, T-cells can become less active and exhausted. This may lead to T-cell death or cause them to change into a type of helper cell (Th2), which creates an environment that suppresses the immune system [84,96,97,98]. T_reg_ cells maintain immunological balance and are prevalent in DLBCL, exhibiting immunosuppressive properties. The impact of T_reg_ cells on the disease outcome is still unclear [99]. FOXP3 is a key marker for T_reg_ cells; high levels of Tim-3+Foxp3+ T_reg_ cells correlate with poor survival in DLBCL, while CD25+FOXP3+ Tregs are linked to better prognosis. CD8+ T-cells, known as CTLs, are essential for directly killing infected or cancerous cells. They play a crucial role in antitumor immunity alongside NK cells. CD8+ tumor-infiltrating lymphocytes (TILs) provide the main antitumor response in DLBCL [99,100]. A low presence of TILs and a high CD4/CD8 ratio are associated with reduced survival, highlighting the importance of CD8+ T-cells. Although PD-1 expression in TILs is linked to shorter survival, a PD-1 blockade with nivolumab has proven ineffective in relapsed/refractory DLBCL patients [101,102].

#### 3.1.3. Natural Killer (NK) Cells

Natural killer (NK) cells are innate lymphoid cells that play a crucial role in fighting tumors. They are capable of distinguishing cancer cells from healthy ones and can activate their cytotoxic functions and produce cytokines by recognizing specific targets. NK cells are also modulators of the interactions with dendritic cells, endothelial cells, macrophages, and T-cells. When NK cell function is disrupted, it can lead to cancer progression through uncontrolled cell growth and metastasis. NK cells also express immune checkpoint molecules like PD-1, with increased levels found in diffuse large B-cell lymphoma. Targeting PD-1 may provide a promising treatment strategy for DLBCL in the future [103,104]. 

### 3.2. Myeloid Cells

#### 3.2.1. Dendritic Cells

Dendritic cells are key players in the tumor microenvironment of diffuse large B-cell lymphoma. These antigen-presenting cells activate naïve T-cells and facilitate their differentiation. A reduction in CD11c+ dendritic cells in the DLBCL TME is a significant unfavorable prognostic factor linked to shorter survival and predicts double- or triple-hit genotypes. In contrast, higher levels of DCs in the TME are associated with better clinical outcomes. Furthermore, increased CD11c+ DC levels in the peripheral blood of DLBCL patients correlate with improved overall survival (OS) [6,105].

#### 3.2.2. Tumor-Associated Macrophages (TAMs)

Tumor-associated macrophages (TAMs) in the tumor microenvironment (TME) play a crucial role in cancer cell survival and progression. They can either kill tumor cells (M1 phenotype) or promote tumor cell survival (M2 phenotype). TAMs are involved in lymphoma pathogenesis and can provide prognostic information for patients with DLBCL [46,106]. Studies indicate that a higher number of CD14+ monocytes with reduced HLA-DR expression correlates with aggressive and refractory disease. Additionally, the overexpression of M2 TAMs at diagnosis is linked to a worse prognosis. An increased ratio of CD163/CD68+ cells independently predicts shorter overall survival (OS) and progression-free survival (PFS). However, some studies have challenged the prognostic significance of TAMs in DLBCL, indicating that CD68 overexpression does not always correlate with poor clinical outcomes. Notably, CD68+ TAM levels have been shown to correlate with shorter OS in patients treated with CHOP chemotherapy, whereas patients receiving chemoimmunotherapy, like Rituximab + CHOP, with higher CD68 expression, had improved OS. Recent research has started to identify the prognostic value of TAMs measured through liquid biopsy, with the findings suggesting that elevated serum soluble CD163 levels are linked to shorter OS in DLBCL patients [6,106,107].

#### 3.2.3. Myeloid-Derived Suppressor Cells (MDSCs) 

Myeloid-derived suppressor cells (MDSCs), originating from immature myeloid cells, are a diverse group of cells that suppress immune responses. They are typically identified through flow cytometry using different markers. There are two main subpopulations, namely monocytic MDSCs (M-MDSCs), which express CD14 and can differentiate into monocytes, and granulocytic MDSCs (G-MDSCs), which express CD15 and can develop into granulocytes. MDSCs primarily inhibit T-cell responses through various mechanisms, including the production of reactive oxygen species and nitrogen oxides, as well as inducing T_reg_s, which aid tumors to spread. They also promote angiogenesis by producing angiogenic factors and proteases [108].

Additionally, MDSCs hinder NK cell maturation and function while polarizing macrophages toward the M2 phenotype by secreting IL-10 and TGF-β. A recent study found elevated levels of M-MDSCs in the liquid biopsies of newly diagnosed or relapsed DLBCL patients [109]. In newly diagnosed patients, a higher frequency of M-MDSCs correlated positively with tumor progression and negatively with overall survival [109,110].

#### 3.2.4. Tumor-Associated Neutrophils (TANs)

Tumor-associated neutrophils (TANs) are a key component of the tumor microenvironment (TME) and exhibit both pro-tumor and antitumor effects. N1 neutrophils have antitumor properties, producing immune-activating cytokines and chemokines that enhance their ability to kill tumor cells. In contrast, N2 neutrophils promote tumor growth by recruiting immunosuppressive CD4+ T-cells and upregulating CCL2, which stimulate angiogenesis [111]. Circulating neutrophils infiltrate tumors and differentiate into T1 and T2 tumor-associated neutrophils (TANs), which can further evolve into a third subset, T3 with a pro-tumoral and proangiogenic effect [112]. About 50% of DLBCL cases show increased levels of neutrophil-derived APRIL, a proliferation-inducing TNF ligand, also known as TALL-2, a proliferation-inducing ligand that may worsen lymphoma aggressiveness. This APRIL is primarily produced by inflammatory neutrophils within DLBCL lesions and can impact the disease outcomes [6,113,114].

#### 3.2.5. Mast Cells (MCs)

Mast cells represent an important component of TME in DLBCL. MCs are recruited to the TME in response to tumor-secreted stem cell factor (SCF), also known as c-KIT ligand, which binds to the c-KIT receptor on MCs, promoting their migration and accumulation. MCs support tumor development by releasing angiogenic mediators, MMPs, and histamine, which facilitate neovascularization. Simultaneously, their secretion of TNF-α and IL-10 promotes regulatory T (Treg) cell-mediated immune tolerance, weakening anti-tumor immune responses and enhancing tumor progression [87]. Studies evaluating MCs in DLBCL have different results. For example, in a study conducted by Hedstrom, MC infiltration was related to a better prognosis, while another study evaluating tryptase expression, secreted by MCs leading to increased levels of VEGF and fibroblast growth factor-2, showed that high levels of tryptase is associated with a poorer response to R-CHOP treatment [87,115,116]. 

### 3.3. Cancer-Associated Fibroblasts

Fibroblasts are mesenchymal cells in connective tissue known for their resilience in stressful conditions. They can differentiate into active fibroblasts, which produce growth factors and help synthesise the extracellular matrix (ECM), and that are different from cancer-associated fibroblasts (CAFs), which contribute to tumor growth by enhancing migration, promoting growth factor signaling, and increasing secretory molecule production. CAFs play a key role in tumor immunity and exist as a diverse and adaptable population within the tumor microenvironment (TME). Distinct CAF subtypes, such as antigen-presenting CAFs (ApCAFs), inflammatory CAFs (iCAFs), and myofibroblast-like CAFs (myCAFs), have been identified, each with unique characteristics and roles in tumor development. Due to CAFs’ complexity and adaptability, most clinicals trials failed, emphasizing the need for more research to better understand their identity and function [117].

### 3.4. Myofibroblasts (MFs)

MFs are stromal cells with an integral component of the lymphoma TME. MFs closely interact with fibroblastic reticular cells (FRCs), a specialized immunomodulatory stromal subset. These stromal cells are influenced by transforming growth factor-beta 1 (TGF-β1), which orchestrates myofibroblast differentiation, fibrotic remodeling, and Treg-mediated immunosuppression, thereby enabling lymphoma cells to evade immune detection. TGF-β1 also promotes the differentiation of CD4+ Th17+ T helper cells, contributing to a proinflammatory axis within the TME [87,118].

### 3.5. Endothelial Cells (ECs)

ECs are integral components of the TME in DLBCL, influencing tumor progression through various mechanisms, like angiogenesis, immune evasion, genetic alterations, and signaling pathway interactions. Angiogenesis is a critical prognostic factor in lymphomas. It also may represent a promising target for next-generation drugs. Tumor-associated blood vessels in DLBCL exhibit a distinct morphology, gene expression profiles, and functional characteristics compared to normal vasculature. In DLBCL, capillaries are composed of two endothelial cell layers with abundant cytoplasm arranged parallel to each other, forming a slit-like lumen [119].

The signal transducer and activator of the transcription-3 (STAT3) pathway plays a key role in driving angiogenesis, tumor cell proliferation, survival, and metastasis. This is mediated, in part, through the regulation of CD163+ macrophages and CD8+ T-cells. *STAT3* activation induces the expression of hypoxia-inducible factor 1-alpha (HIF-1α) and vascular endothelial growth factor (VEGF) in tumor cells, while VEGF, in turn, activates *STAT3* in endothelial cells, establishing a feed-forward loop that sustains angiogenesis. Moreover, *STAT3* inhibits the expression of the tumor suppressor, p53, thereby further promoting tumor neovascularization.

Additionally, the overexpression of sphingosine kinase 1 (SPHK1), an enzyme responsible for the production of sphingosine-1-phosphate (S1P), serves as a potent driver of angiogenesis in DLBCL, by activating endothelial cells which results in additional STAT3 activation and angiogenesis in the tumor microenvironment [87,120,121]. 

### 3.6. Extracellular Matrix (ECM)

The ECM plays a pivotal role in the tumor microenvironment (TME) of diffuse large B-cell lymphoma (DLBCL), influencing tumor progression, metastasis, and therapeutic response. The ECM is a dynamic network of proteins and proteoglycans that provides structural support and regulates cellular functions. In DLBCL, ECM remodeling is characterized by alterations in the protein content and enzymatic activity, impacting signal transduction and cell–matrix interactions. This remodeling is induced by factors such as hypoxia, acidosis, inflammatory cells, or proteases secreted by a tumor or stromal cells [122].

Related to ECM, Lenz et al. [123] described two new gene expression profiles of DLBCL. These two groups were associated with different prognoses in patients treated with R-CHOP. The two groups are classified as “Stromal-1 signature” and “Stromal-2 signature”. The stromal-1 signature is found in tumors with high macrophage and extracellular matrix components’ infiltration. This subtype encodes various types of collagen, laminin isoforms, fibronectin, osteonectin, connective-tissue growth factor (CTGF), and the antiangiogenic factor, thrombospondin. More than that, modifiers of collagen synthesis, like LOXL1 and SERPINH1, change and remodel proteins like MMP2, MMP9, MMP14, PLAU, and TIMP2. The “stromal-1 signature” in diffuse large B-cell lymphoma (DLBCL) comprises genes predominantly expressed in the cells of the monocytic lineage, including those encoding key transcription factors and cytokine receptors. Among these, CEBPA encodes CCAAT/enhancer-binding protein alpha (C/EBPα), a transcription factor involved in myeloid differentiation, while CSF2RA encodes the colony-stimulating factor 2 receptor subunit alpha, which plays a role in hematopoietic cell proliferation and survival. Additionally, osteonectin, known as a secreted protein acidic and rich in cysteine (SPARC), is highly expressed by TAMs within the lymphoma TME. SPARC plays a complex role in lymphoid malignancies, acting either as a tumor suppressor by inhibiting cell proliferation and inducing apoptosis, or as a tumor promoter by enhancing extracellular matrix (ECM) remodeling, angiogenesis, and immune evasion. The dual nature of SPARC highlights its context-dependent function in lymphoma progression and response to therapy. “Stromal-2 signature” is characterized by the expression of endothelial cell markers, including CD31, also known as platelet endothelial cell adhesion molecule-1, PECAM-1, and von Willebrand factor (VWF), both of which are essential for vascular integrity and endothelial function. Additionally, this signature includes genes specifically expressed in endothelial cells, such as EGFL7 (Epidermal Growth Factor-Like Domain 7), MMRN2 (Multimerin 2), GPR116 (G Protein-Coupled Receptor 116), and SPARCL1 (SPARC-like protein 1), which contribute to endothelial signaling, vascular homeostasis, and angiogenesis. Furthermore, the “stromal-2 signature” encodes the key regulators of angiogenesis, VEGF. In addition to endothelial-related genes, this signature also includes genes that are exclusively expressed in adipocytes, such as ADIPOQ (Adiponectin), FABP4 (Fatty Acid Binding Protein 4), RBP4 (Retinol Binding Protein 4), and PLIN (Perilipin). These adipocyte-associated factors may contribute to the metabolic reprogramming within the tumor microenvironment, influencing lymphoma progression and the therapeutic response [122,123].

## 4. Epigenetic Influence on the Tumor Microenvironment

### 4.1. Epigenetic Modification of the TME 

Most research on cancer has focused on the epigenetic alterations in cancer cells. However, recent studies show that these modifications also impact the immune cells in the tumor microenvironment (TME). Investigating the effects of epigenetic changes on the immune cell function in the TME has become increasingly important [20]. Epigenetic modifications affect lymphoid cells. For example, DNA methylation by DNMT1 and H3K27 trimethylation by *EZH2* hinder T-cell infiltration in the TME. Exhausted CD8+ T-cells demonstrate different DNA methylation patterns, such as the methylation of LAG3 in naive cells and its demethylation during CD8+ T-cell activation. LSD1, a lysine-specific demethylase, also influences T-cell infiltration; inhibiting LSD1 enhances H3K4me2 levels and promotes CD8+ T-cell recruitment, as seen in breast cancer. Treg-specific gene expression relies on DNA hypomethylation, and *EZH2* inhibition has been associated with Treg pro-inflammatory activity [124]. In NK cells, epigenetic changes affect maturation, differentiation, and activation. NK cell surface receptors are regulated epigenetically, linking epigenetics to NK cell tumor activity. Histone acetylation controls activating receptors like NKp30 and NKp46. The NKG2D gene remains unmethylated in NK cells and is linked to high H3K9 acetylation. Increased NKG2D receptor levels correlate with decreased H3K27me3 due to *EZH2* inhibition [124,125]. While lower levels of activating receptors may suggest NK cell exhaustion, studies show that enhanced expansion and cytotoxicity of NK cells are associated with increased NKG2D. Tumor-associated macrophages (TAMs) correlate with a poor prognosis in lymphomas, leading to investigations into TAM polarization. Epigenetic modifications have identified factors that regulate M2 polarization. DNMT3B downregulation increases M2 markers like Arg1 [126,127]. Changes in H3K4 and H3K27 methylation regulate M2 marker expression. Key proteins include H3K4 methyltransferase SET, SMYD3, and JMJD3, which promote M2 polarization. Certain HDACs, like HDAC4 and SIRT2, are linked to M2 phenotype regulation, particularly in Arg1 expression. Conversely, HDAC3 and HDAC9 negatively regulate M2 polarization, with the knockdown of HDAC3 reducing inflammation and HDAC9 depletion downregulating inflammatory genes [128]. In myeloid-derived suppressor cells (MDSCs), HDACs regulate their immunosuppressive functions. MDSCs lacking HDAC11 show increased suppressive activity in the TME; this is related to higher ARG1 and Nos2 levels. Inhibiting class I histone deacetylases with Entinostat reduces the immunosuppressive functions of monocytic MDSCs by lowering ARG1, iNOS, and COX-2 levels. HDAC11 may also regulate IL-10 levels and MDSC expansion, while HDAC6 could influence IL-10 expression as a transcription factor, although this remains unproven in lymphomas. Additionally, certain microRNAs, particularly miR494, are upregulated in MDSCs from various tumor models, including B-cell lymphomas [129,130,131,132]. Looking at TANs, we see that the balance between pro-tumor (N2) and anti-tumor (N1) phenotypes is dictated by changes in DNA methylation affecting TAN differentiation, histone modifications regulating pro- and anti-inflammatory gene expression, and ncRNAs influencing TAN-mediated immune suppression. Hypermethylation of immune-activating genes in neutrophils suppresses the N1 phenotype and enhances N2 phenotype. TET2 downregulation in neutrophils promotes the accumulation of immunosuppressive N2 TANs, which contribute to lymphoma progression [88].

Hypermethylation of IFN-γ signaling genes, JAK1, JAK2, and STAT1 reduces N1 polarization, impairing neutrophil-mediated tumor control [25]. *EZH2* catalyzes H3K27me3, repressing pro-inflammatory and anti-tumor genes in TANs; therefore, *EZH2* inhibition can restore neutrophil-mediated tumor cytotoxicity [133].

Epigenetic modifications also influence stromal function in two ways, via the activation of CAFs and ECM remodeling and immune modulation. The hypermethylation of CDKN2A (p16) promotes uncontrolled fibroblast proliferation and RASSF1A silencing via DNA methylation which prevents apoptosis and plays a important role in cell-cycle arrest and is implicated in the development of a number of different cancers including lymphomas [134,135]. Epigenetic reprogramming in cancer-associated fibroblasts (CAFs), characterized by reduced histone methylation and increased histone acetylation, facilitates their activation and supports tumor development. Transforming growth factor-β (TGFβ) and other CAF-activating stimuli contribute to these chromatin modifications. [136]. *EZH2*-mediated H3K27me3 repression of anti-fibrotic genes leads to a fibrotic, desmoplastic TME, supporting tumor survival [137]. Regarding ncRNAs, miR-221 upregulation from CAFs enhances pro-tumorigenic cytokine secretion, fostering a lymphoma-permissive microenvironment, and lncRNA HOTAIR overexpression induces epigenetic remodeling, facilitating CAF expansion and matrix remodeling [138,139]. Looking at ECM, epigenetic modification alters stiffness and fibrosis, which results in ECM accumulation, promoting a rigid tumor-supportive stroma, and histone deacetylation in stromal cells reduces ECM turnover, making the microenvironment more fibrotic and resistant to therapy [123,140].

### 4.2. Epigenetic Modulation of the TME

Epigenetic modification plays an important role in shaping the immune evasion mechanisms within the TME. Immune evasion allows lymphoma cells to escape immune surveillance by downregulating antigen presentation, modifying immune checkpoint signaling, and reprogramming the tumor immune landscape. Understanding these epigenetic alterations provides novel therapeutic opportunities, particularly in the combination of epigenetic drugs with immune checkpoint inhibitors (ICIs) or CAR-T therapy.

#### 4.2.1. DNA Methylation and Antigen Presentation Suppression

The DNA methylation of the genes involved in antigen presentation allows malignant cells to evade immune detection, contributing to tumor progression and resistance to immunotherapy. Methylation-mediated immune evasion mechanisms in DLBCL primarily affect MHC molecules, costimulatory molecules (CD80/CD86), antigen processing machinery (TAP1/TAP2, B2M), and cytokine and interferon signaling pathways. The Class II Transactivator (CIITA) is a master regulator of MHC-II expression in antigen-presenting cells and B-cells. In DLBCL, the hypermethylation of the CIITA promoter leads to its silencing, resulting in a loss of MHC-II expression. Also, reduced MHC-II expression prevents tumor cells from presenting antigens to CD4+ T-cells, impairing helper T-cell activation and weakening the adaptive immune response [141]. The DNA methylation of B2M and MHC-I downregulation is another mechanism described in DLBCL. β2-microglobulin (B2M) is essential for the stability and function of MHC-I molecules, which present antigens to CD8+ cytotoxic T-cells. Promoter methylation of B2M leads to the downregulation or complete loss of MHC-I expression, allowing lymphoma cells to escape T-cell-mediated killing. B2M mutations and deletions are also common in DLBCL and work synergistically with epigenetic silencing to reduce MHC-I antigen presentation [142]. The epigenetic suppression of Antigen-Processing Machinery is modulated by TAP1 and TAP2, crucial transporters that load peptides onto MHC-I molecules for antigen presentation. The hypermethylation of TAP1/TAP2 genes decreases their expression, further impairing antigen presentation and weakening CD8+ T-cell responses. Restoring TAP expression using DNMT inhibitors can reverse the immune escape and enhance tumor recognition by cytotoxic T-cells [143]. CD80 and CD86 are essential costimulatory molecules that interact with CD28 on T-cells, providing activation signals for an effective immune response. Promoter hypermethylation of CD80/CD86 genes suppresses their expression, reducing T-cell activation and promoting immune tolerance. This leads to a dysfunctional immune response, allowing lymphoma cells to proliferate unchecked [144]. PD-L1 upregulation and T-cell exhaustion it is another mechanism by which DNA methylation influences the TME. PD-L1, a negative immune checkpoint regulator is frequently upregulated in DLBCL via epigenetic mechanisms. The hypomethylation of the PD-L1 promoter, combined with histone modifications, leads to its overexpression, contributing to T-cell exhaustion and immune suppression [145,146]. Interferon-gamma (IFN-γ) is a key cytokine that enhances antigen presentation by inducing MHC expression. The hypermethylation of IFN-γ-responsive genes, such as JAK1, JAK2, and STAT1, reduces their expression, silencing the IFN-γ signaling pathway and further suppressing antigen presentation. This prevents immune cells from mounting an effective anti-lymphoma response [12]. Interleukin-12 (IL-12) promotes T_h1_ immune responses, which are crucial for anti-tumor immunity. Hypermethylation of IL-12B suppresses its expression, reducing T_h1_ responses and shifting the TME toward an immune-suppressive T_h2_ phenotype, that favors tumor progression and limits the effectiveness of immune checkpoint blockade therapies [12].

#### 4.2.2. Histone Modifications and Checkpoint Inhibitor Regulation

Histone modifications regulate checkpoint molecule expression, affecting T-cell exhaustion. For example, the acetylation of H3K27ac at the PD-L1 promoter enhances its expression, inhibiting T-cell activity, correlating with a poorer prognosis; therefore, HDACi can reduce PD-L1 expression, restoring the immune function. *EZH2* overexpression in DLBCL leads to the H3K27me3-mediated suppression of the genes involved in antigen presentation, including CIITA, TAP1, and B2M [145]. This results in reduced MHC expression, impairing the ability of T-cells to recognize and attack tumor cells. Also, *EZH2* silences CXCL9 and CXCL10, which are T-cell recruitment chemokines, further contributing to a T-cell-excluded tumor microenvironment [12]. 

#### 4.2.3. ncRNAs and Immune Escape

miR-155 is one of the most well-characterized oncogenic miRNAs in hematological malignancies, including DLBCL. It is upregulated in tumor cells and immune cells within the TME, where it suppresses anti-tumor immune responses. *SOCS1* is a negative regulator of the JAK/STAT signaling pathway, which controls inflammation and immune responses. miR-155 silences *SOCS1*, leading to the persistent activation of JAK/STAT signaling. This results in an immunosuppressive microenvironment, increasing the levels of immunoregulatory cytokines such as IL-10 and TGF-β, which suppress cytotoxic T-cell activity [147]. miR-155 it is also responsible for the recruitment of T_regs_, that are essential for maintaining immune tolerance, but in cancer, they promote immune evasion by suppressing anti-tumor immune responses. The upregulation of miR-155 increases T_reg_ infiltration into the TME, further reducing the T-cell function. The inhibition of miR-155 in preclinical models has been shown to reduce T_reg_ accumulation and restore T-cell cytotoxicity against lymphoma cells [148].

One of the most studied oncogenic lncRNAs in DLBCL is Metastasis-Associated Lung Adenocarcinoma Transcript 1 (MALAT1), which plays a key role in modulating tumor-associated macrophages (TAMs). lncRNA MALAT1 promotes M2 macrophage differentiation, leading to an immune-suppressive tumor microenvironment, by upregulating IL-10 and TGF-β, which suppress cytotoxic T-cell responses and downregulating pro-inflammatory cytokines (IL-12, TNF-α), reducing the ability of macrophages to activate T-cells [149,150].

## 5. Targeting Epigenetics in DLBCL

### 5.1. Targeting DNA Methylation

The Food and Drug Administration (FDA) has approved Azacitidine and Decitabine as the first DNA methyltransferase inhibitors (DNMT inhibitors or DNMTis) for clinical application, with no important efficacy and a very high toxicity profile for myelodysplastic syndrome (MDS) and acute myeloid leukemia (AML) [151,152]. In recent years, there has been a notable increase in clinical trials that demonstrate the efficacy of DNMT inhibitors in the treatment of B-NHLs [153]. One significant preclinical study indicated that the combination of azacitidine or decitabine with Vorinostat markedly improved antitumor activity in patients with relapsed or refractory DLBCL. Although a subsequent phase Ib clinical trial did not produce substantial clinical outcomes, it suggested a potential for prolonged chemosensitivity, indicating that these agents might assist in preserving the treatment effectiveness over time [153]. A study of patients with relapsed malignant lymphomas used oral decitabine along with another drug called tetrahydrouridine that blocks CDA. This combination resulted in an overall response rate (ORR) exceeding 30%, underscoring its potential efficacy in this patient demographic [154]. Additionally, a phase III clinical trial conducted in 2022 evaluated the effectiveness of azacitidine when used alongside the established R-CHOP regimen for untreated intermediate- to high-risk DLBCL or transformed follicular lymphoma. This trial reported an impressive ORR of 94.9%, with 88.1% of participants achieving a complete response (CR). Such findings suggest that the integration of azacitidine can improve the therapeutic efficacy of the R-CHOP regimen for B-cell lymphoma. It is a phase II/III randomized controlled trial that is investigating the combined use of azacitidine and R-CHOP, specifically in elderly NHL patients over 75 years old. This study aims to validate the hypothesis that adding azacitidine to this treatment regimen could extend the progression-free survival (PFS), thereby reinforcing the potential role of DNMT inhibitors in lymphoma treatment [155,156]. Combining DNMTis with other therapeutic agents has emerged as a promising strategy to enhance the treatment efficacy in DLBCL. Study NCT06158399 is a phase 2 trial evaluating the combination of Azacitidine with an R-CHOP regimen in *TP53*-mutated untreated DLBCL, regimen ARCHOP. Another recruiting study, NCT05823701, is combining Azacitidine with Chidamide, and Obinutuzumab and mitoxantrone liposome in patients with relapsed and refractory DLBCL. Chidamide is a class I HDACi approved by the FDA for R/R peripheral T-cell lymphoma. The trial, Javelin DLBCL, NCT02951156, evaluated Avelumab, an anti-PD-L1 agent and Utomilumab, a fully human IgG2 CD 137/4-1BB agonist in different combinations with Rituximab, Azacitidine, and Bendamustine, versus the investigator’s choice of Rituximab and Bendamustine or Rituximab, Gemcitabine, and Oxaliplatin. The study was discontinued before the initiation of phase 3 due to the closure of study arms following futility analysis and difficulty in enrolling participants due to the evolving treatment landscape [157].

Decitabine in clinical trial, NCT03579082, evaluated the safety, tolerability, and clinical effects of Decitabine combined with R±DHAP for patients with R/R DLBCL. The results of this trial were an ORR 40% for decitabine–RDHAP and 33% in the RDHAP groups, with no difference between the groups. DOR for the decitabine–RDHAP regimen was higher than that for the control regimen (*p* = 0.044) and after a median follow-up of 12.0 months, the median PFS was 7 months in the decitabine–RDHAP group and 5 months in the control group. The OS was 17 months for the decitabine–RDHAP group and 9 months in the RDHAP group with no significant differences between the two groups (*p* = 0.47, 0.17). The incidence of adverse events was not significantly different between the groups [158]. Decitabine is also being evaluated in another trial with the purpose of evaluating the efficacy and safety of low-dose decitabine combined with Cyclophosphamide/Vindesine/Bonisone (COP), now known as the regimen D-COP for R/R DLBCL. This study’s rationale is related to the greater cardiac toxicity associated with adriamycin, that made the COP regimen widely used in patients with DLBCL with a good response rate and due to the high side effects of high-dose decitabine with a poor patient tolerance. No data have been published yet. The study of DNA methylation is very important, given its integral role in tumor development and progression. The study of DNA methylation patterns not only aids in refining the tumor subtype classification and personalizing the treatment approaches for patients with B-NHLs but also enhances the identification of novel therapeutic targets. All this research contributes to the development of more effective targeted therapies and combinations of treatments, bringing forward the significant clinical importance of investigating aberrant DNA methylation in B-NHLs [46].

### 5.2. Targeting Histone Methylation

The current focus on the development of histone methylation inhibitors primarily targets *EZH2*. Clinical trials involving various *EZH2* inhibitors, such as valemetostat, tazemetostat, and others, have shown promising therapeutic potential [159,160]. Generally, *EZH2* inhibitors exert antitumor effects indirectly by influencing the associated proteins and signaling pathways. The experimental findings suggest that novel *EZH2* inhibitors, like ribavirin and IHMT-EZH2-115, may hold promise against B-cell lymphomas [161,162]. Clinical trials have reported encouraging results with *EZH2* inhibitors. For example, one trial demonstrated efficacy in relapsed/refractory B-cell non-Hodgkin lymphomas (B-NHLs), and a phase I trial of tazemetostat monotherapy achieved an overall response rate (ORR) of 57% (four out of seven patients) [163,164]. In a phase II trial for follicular lymphoma (FL), the experimental group with *EZH2* mutations showed a higher ORR and progression-free survival (PFS) compared to the control group without *EZH2* mutations [165]. A trial involving DLBCL, which combined Tazemetostat with atezolizumab, also resulted in a 57% ORR in four out of seven patients [166]. In another trial that incorporated Tazemetostat into the standard R-CHOP regimen for DLBCL treatment yielded promising results, highlighting the potential of histone methylation combination therapies [167]. NCT02889523 is a phase 1 active trial that is evaluating Tazemetostat in newly diagnosed DLBCL and FL patients treated with chemotherapy, the Epi-RCHOP regimen. NCT02875548 is another active trial, that completed the recruitment of patients with the aim to evaluate the long-term safety of Tezemetostat, by providing continuing access to Tazemetostat for people that have previously completed participation in a Tazemetostat study, either with monotherapy or combination therapy. While *EZH2* inhibitors pave the way for innovative combination therapies for B-NHLs, their long-term efficacy still requires validation through extensive clinical studies.

### 5.3. Targeting Histone Acetylation

Advancements in histone acetylation research have led to new drugs and therapies, particularly histone deacetylase inhibitors (HDACis). These include classes such as benzene carbamides, cyclic peptides, fatty acids, and oxalates [168]. Research highlights the effectiveness of HDACis in treating lymphomas. Notably, Pracinostat shows significant antitumor activity in DLBCL, with varying responses among tumor subtypes [169]. In a 2018 phase I trial, combining valproate with R-CHOP in DLBCL patients resulted in 84.7% progression-free survival (PFS) and 96.8% overall survival (OS) over two years [170]. A 2019 phase I–II trial of vorinostat, cladribine, and Rituximab in relapsed B-NHL and untreated MCL reported median PFS and OS of 5.95 months and 10.95 months, respectively [170]. Furthermore, combining Tucidinostat with R-CHOP led to a 94% overall response rate, including an 86% complete response rate. Vorinostat was also evaluated in combination with the R-CVEP regimen in elderly patients with R/R DLBCL. The aim of this trial was to replace procarbazine, a drug with many side effects with a new novel drug, Vorinostat, in a drug combination for the treatment of DLBCL patients [171]. A 2020 study showed that Tucidinostat, combined with the R-CHOP regimen, is effective for elderly DLBCL patients. Its use with Rituximab enhances CD20 upregulation and inhibits tumor growth. Clinical trials confirm the safety and efficacy of HDACi drugs [172,173]. A 2021 study found that Tucidinostat induces apoptosis in DLBCL cells by targeting the HDACs/STAT3/Bcl-2 pathway, while Abexinostat has proven effective in relapsed or refractory follicular lymphoma (FL) and mantle cell lymphoma (MCL). HDACis are also being explored in combination therapies [174]. Another active study, still recruiting at this time, NCT06779435, is aimed to evaluate the real-world data of regimens containing Tucidinostat for the primary treatment of DLBCL. It is a prospective, observational, multicenter, cohort study with 400 newly treated DLBCL patients who will be divided into two groups, namely the Unfit group which will include patients over 80 years old or younger but with comorbidities that will receive a combination regimen including C-R2 and C-R-mini-CHOP, and the group that cannot be defined as unfit which will receive a combination regimen including CR-CHOP, C-Pola-R-CHP, etc. Despite these successes, the efficacy of HDACi monotherapy in B-NHLs is limited, with trials of Vorinostat and Mocetinostat showing mixed results and cases of resistance. Understanding the HDACi resistance mechanisms and integrating HDACis with other immunotherapies will be key to future lymphoma treatments [46]. A study published in 2023 evaluating the efficacy of Mocetinostat in patients with R/R FL or DLBCL and *CREBBP* or *EP300* mutations, included seven patients in the trial, showed a median PFS of 4.6 months and similar median EFS [174]. 

### 5.4. Targeting miRNAs 

Various drugs targeting microRNAs (miRNAs) show promising antitumor effects in therapeutic settings. For instance, Bortezomib, a proteasome inhibitor, targets miR-198 to suppress HMGA1 expression, which in turn inhibits the progression of DLBCL [175]. Additionally, the oligonucleotide inhibitor, Cobomarsen, hinders DLBCL growth by targeting miR-155 [176]. Research has shown that miR-21 can influence the effectiveness of the first-line treatment regimens for DLBCL by affecting the PI3K/Akt signaling pathway [177]. Curcumin has been found to induce the downregulation of miR-21 and upregulation of VHL in DLBCL, promoting tumor suppression and apoptosis [178]. MRX34, a double-stranded miR-34 mimic developed by Mirna Therapeutics, has recently completed clinical trials and communicated data in chronic lymphocytic leukemia (CLL) [179,180]. The results indicated a manageable safety profile and preliminary efficacy. Overall, these findings suggest that drugs targeting miRNAs hold significant promise as novel therapeutic strategies for this type of lymphoma.

### 5.5. Targeting Chromatin Remodeling

Mutations in BCL7A, commonly observed in diffuse large B-cell lymphoma, can disrupt its binding to the SWI/SNF complex, which impairs tumor suppression. Additionally, mutations in the SWI/SNF complex may affect the effectiveness of treatments like ibrutinib combined with Venetoclax [181,182,183]. Research data communicated on chromatin remodeling has offered some light on the mechanisms behind the drug resistance in tumor cells. Drug resistance often stems from changes in chromatin remodeling and gene expression. Therefore, targeting the pathways related to chromatin remodeling could be an effective strategy for improving the prognosis of lymphoma patients.

### 5.6. Immune Checkpoint Inhibitors in DLBCL

Over the last ten years, immune checkpoint inhibitors (ICIs) targeting PD-1, PD-L1, and CTLA-4 have demonstrated significant efficacy in treating various solid tumors and hematologic malignancies, like Hodgkin lymphoma, and there was considerable optimism that similar results will be obtained in DLBCL. PD-1 is an inhibitory co-receptor expressed on B-cells, NK, CD4+ and CD8+ T-cells, and tumor-infiltrating lymphocytes (TILs) (1). On B-cells, PD-1 is regulated by BCR. PD-1 interacts with PD-L1, expressed on the cell surfaces of activated B-, NK and T-cells, peripheral tissues and organs, and tumor cells, and with PD-L2, expressed by macrophages and DCs [184,185,186]. In lymphomas, the highest level of PD-L1 expression has been observed in DLBCL, in non-malignant cells in 25–75% of cases [187,188]. There are two anti-PD-1 agents, Nivolumab and Pembrolizumab and three anti-PD-L-1 agents, Atezolizumab, Avelumab, and Durvalumab, approved for the treatment of lymphomas. Nivolumab, an anti-PD-1 agent that induces the proliferation of lymphocytes and the release of IFN-y, in an initial Phase I study evaluating immune checkpoint inhibitors in patients with relapsed/refractory (R/R) follicular lymphoma (FL) or diffuse large B-cell lymphoma (DLBCL) demonstrated an overall response rate (ORR) of approximately 40%. Additionally, the treatment exhibited a manageable toxicity profile, suggesting a potential clinical benefit [189]. The same data were obtained in the phase II trial, CheckMate 139 [190]. Pembrolizumab binds with high affinity to human PD-1, blocking receptor ligation by both PD-L1 and PD-L2 and leading to enhanced T-lymphocyte immune responses [191]. Pembrolizumab is now evaluated in a phase 2 trial, NCT03990961, that is evaluating this drug in R/R DLBCL with PD-L1 genetic alterations. Pembrolizumab was also evaluated in R/R DLBCL patients after ASCT, but the results were disappointing, the trial not meeting the primary endpoint PFS compared with ASCT alone [192]. The keynote-013 trial that included a cohort of R/R DLBCL patients did not report any results related to DLBCL patients. However, despite these promising early results, subsequent trials have shown limited efficacy as single agents. Clinical trials of ICIs in the setting of DLBCL have yielded disappointing results thus far. These findings highlight the need for a deeper understanding of the molecular mechanisms underlying the intrinsic immune resistance in these lymphomas. Identifying these molecular determinants is essential for developing more effective combination strategies that can overcome these results [193]. Tafasitamab monotherapy has demonstrated clinical activity in patients, including R/R DLBCL, as observed in a phase II study. Across different subtypes, the response rates ranged from 20% to 30%, with an ORR of 25.7% in 9 out of 35 DLBCL patients. Among these responders, seven achieved a partial response, while two had a complete response. The median duration of response was 20.1 months [194].

Tafasitamab is a humanized monoclonal antibody that specifically targets CD19 and has recently received FDA approval for use in combination with lenalidomide in treating R/R DLBCL in patients ineligible for ASCT. A key feature of Tafasitamab is its engineered Fc region, which has been modified to enhance the binding affinity to Fcγ receptors, amplifying the immune response against malignant B-cells [195].

### 5.7. Chimeric Antigen Receptor (CAR) T-Cells

CAR-T therapy is a type of immunotherapy where a patient’s T-cells are genetically engineered to express a Chimeric Antigen Receptor (CAR). This receptor allows T-cells to specifically recognize and attack cancer cells. CD19-targeting CAR T-cells have gained significant attention, showing long-term, durable efficacy in patients with DLBCL and a very poor prognosis [196,197]. There are three CD19-targeting CAR T-cell therapies that are currently approved for DLBCL. As previously experienced with immunotherapy, CAR T-cell therapy has brought its own set of toxicities, that are unique with cytokine release syndrome (CRS) and neurotoxicity being very common and deserving specific attention due to the severity [196]. 

Axicabtagene ciloleucel (axi-cel), Yescarta, is a CD19-targeting CAR T-cell therapy that stands out from other approved treatments due to its CD28 costimulatory intracellular domain. This unique feature influences its activation and persistence. The therapy has been approved for the treatment of diffuse large B-cell lymphoma (DLBCL). The ZUMA-1 trial evaluated axi-cel in patients with relapsed or refractory (R/R) DLBCL who had undergone at least two prior lines of therapy. The study reported an overall response rate (ORR) of 82%, with 54% achieving a complete response (CR). Some patients with CR experienced long-term durable responses. However, this treatment was associated with severe side effects, including grade 3–4 cytokine release syndrome (CRS) in 13% of patients and neurotoxicity in 28% [198]. These encouraging results led to axi-cel’s approval for R/R DLBCL patients previously treated with two lines of therapy. More recently, axi-cel showed preliminary efficacy in untreated patients with double- or triple-hit DLBCL or those with positive PET-CT scans following two cycles of a rituximab–anthracycline regimen. The study reported a 93% ORR, including 80% CR, with grade 3–4 CRS in 20% and neurotoxicity in 27% of patients [199]. While these findings are promising, further long-term data are needed to establish axi-cel’s role in untreated DLBCL.

Lisocabtagene maraleucel (liso-cel), Breyanzi, is another CAR T-cell therapy that utilizes a CD3-4-1BB signaling domain and is administered at a fixed CD4:CD8 ratio. In patients with relapsed or refractory (R/R) DLBCL who had received at least two prior lines of therapy, liso-cel achieved an overall response rate (ORR) of 75%, with 55% achieving a complete response (CR). These findings led to its regulatory approval in 2021 for this patient population [200].

Tisagenlecleucel (tisa-cel), Kymriah, is a CAR T-cell therapy incorporating a CD3-4-1BB signaling domain. In patients with relapsed or refractory (R/R) DLBCL who had undergone two or more prior lines of treatment, it achieved an overall response rate (ORR) of 52%, with 40% achieving complete remission (CR). The treatment was associated with a higher incidence of grade 3–4 cytokine release syndrome (CRS) at 22%, while neurotoxicity occurred in 12% of patients, similar to other CAR T-cell therapies. These results led to tisa-cel’s approval for R/R DLBCL [201]. Although CAR T-cell therapy has revolutionized the treatment of B-cell lymphomas, there are still critical areas that require refinement, for example preventing CRS and neurotoxicity that continue to be major concerns, and the integration of CAR T-cell therapy into earlier treatment stages remains an open question requiring further investigation.

### 5.8. Bispecific Antibodies

Bispecific antibodies provide an alternative approach to immune system engagement in lymphomas by utilizing dual antigen-binding sites—one targeting tumor antigens such as CD19 or CD20 in GCB lymphoma, and the other binding to a TCR-activating receptor like CD3. The toxicity profile of bispecific antibodies differs from that of conventional chemoimmunotherapy.

Blinatumomab, a CD3/CD19 bispecific antibody, has been evaluated in patients with R/R DLBCL. In a phase I trial involving previously treated B-cell lymphoma patients, the therapy demonstrated an ORR of 55%, with 36% achieving complete remission [202].

Epcoritamab, a CD3/CD20 bispecific antibody administered subcutaneously, has generated considerable interest due to its convenient delivery method. Its toxicity profile is comparable to that of other bispecific antibodies, and it has demonstrated efficacy in previously treated DLBCL, achieving a 67% ORR and a 33% CR rate. Notably, in patients who had previously received CAR T-cell therapy, the ORR was 100%, with a CR rate of 50%. 

Glofitamab, a CD3/CD20 BiTE with a unique 2:1 antigen-binding configuration, is designed to enhance tumor antigen targeting. To mitigate toxicity, in the first studies it has been administered in combination with Obinutuzumab. The ORR was 53.8% and CR was 36.8%. Of 63 patients that had CR, 84.1% had durable, ongoing CR after 27.4 months of observation. 

CRS occurred in 50.3% patients, with grade 3 or 4 in 3.5%, while 1.2% of patients experienced grade 3 neurotoxicity [203].

Mosunetuzumab, a CD3/CD20-targeting bispecific antibody, incorporates the Fc region to more closely mimic the structure of human antibodies. It is currently under investigation for use in previously treated B-cell lymphomas and has shown promising results. In clinical studies of DLBCL, it achieved an ORR of 33% with a CR rate of 21% [204]. One key advantage of Mosunetuzumab is its manageable toxicity profile, as no grade 3–4 CRS events were reported in trials. Preliminary data have also emerged in previously untreated DLBCL patients who were ineligible for frontline chemotherapy due to their age or comorbidities. In this subset, Mosunetuzumab demonstrated a 55% ORR, with 46% achieving CR. Notably, toxicity remained manageable, with all CRS cases being grade 1. Given the limited treatment options for patients who cannot receive chemotherapy, Mosunetuzumab may offer a viable therapeutic alternative [205].

Odronextamab, another IgG4-based bispecific antibody targeting CD3 and CD20, has demonstrated efficacy in previously treated B-cell lymphomas, including patients who were refractory to CAR T-cell therapy [206].

### 5.9. Antibody-Drug Conjugates (ADCs)

Immunoconjugates, also known as antibody-drug conjugates (ADCs), consist of a monoclonal antibody linked to a cytotoxic agent, referred to as the payload. These payloads are highly potent molecules that would be excessively toxic in their untargeted form but, when attached to an antibody, allow for selective tumor targeting. The ADC binds to a specific antigen on the tumor cell surface, leading to internalization and subsequent payload release, which induces cytotoxicity and tumor cell death. Polatuzumab vedotin (PolaV), an ADC that specifically targets CD79b, comprises a monoclonal antibody linked to monomethyl auristatin E (MMAE), a potent cytotoxic agent. Upon binding to CD79b, the chemical linker is cleaved, releasing MMAE inside the B-cell, where it exerts antimitotic effects and induces apoptosis [207]. In adult patients with R/R DLBCL who are ineligible for hematopoietic stem cell transplantation, PolaV was approved by the European regulatory authority as a second-line treatment in combination with Bendamustine and Rituximab. The FDA granted accelerated approval for PolaV in June 2019, also in combination with BR, for patients with refractory DLBCL following at least two prior therapies [208]. Beyond its direct cytotoxic effects, PolaV significantly influences the tumor immune microenvironment. By eliminating CD79b-expressing B-cells, PolaV may reduce tumor-induced immune suppression and enhance tumor antigen presentation to immune cells. This could trigger a cascade of immune activation events, including increased T-cell recruitment and improved tumor recognition, ultimately leading to a stronger and more sustained anti-tumor immune response [120]. There is growing evidence supporting the combination of PolaV with immune-stimulating antibodies or immune checkpoint inhibitors. These combinations aim to leverage PolaV’s immunomodulatory effects to further enhance the treatment response rates and prolong disease control. Investigating how immune cells interact with PolaV within the tumor microenvironment could provide valuable insights into novel immune based therapeutic strategies aimed at improving long-term patient outcomes [209].

### 5.10. Rationale for Novel Immunotherapy-Based Combinations in B-Cell Lymphomas

The rapid advancement of therapy in DLBCL has introduced exciting new possibilities for these patients. However, there is still substantial room for improvement. As previously discussed, ICIs have demonstrated limited efficacy as a monotherapy but have shown enhanced effectiveness when combined with other treatments. However, the optimal combination strategies and ideal drug partners remain to be identified. While CAR T-cell therapy has led to durable responses in some patients with high-risk and refractory disease, these cases represent only a small subset of patients, and long-term efficacy challenges persist.

Likewise, the sustained effectiveness of bispecific antibodies remains an open question, and various combination strategies are currently under investigation in this area. Having this in mind, it was natural that the combination of different drugs would develop. A major barrier to successful immunotherapy in lymphomas is treatment resistance, which may stem from an immunosuppressive TME and the immune evasion mechanisms employed by these malignancies. Data of clinical trials that are ongoing or under evaluation were collected from clinicaltrial.gov. Representative clinical trials targeting epigenetic modifications in DLBCL are shown in Table 2.

A study of 5-azacitidine in combination with Vorinostat in patients R/R DLBCL showed an ORR of 6.7% and 3 months OS of 77% [149]. Study NCT05816746 is a phase 2 active study evaluating Decitabine and Anti-PD-1 in R/R DLBCL and it is still recruiting. Another active study, NCT06683885, is a phase 1 and 2 prospective trial of the combination therapy of Obutinib or Decitabine with Rituximab, Cyclophosphamide, and Prednisone for the primary treatment of elderly patients with newly diagnosed DLBCL. As mentioned before, the Trial Javelin DLBCL, NCT02951156, evaluated the combination of Avelumab and Utomilumab, in different combinations with Rituximab, Azacitidine, and Bendamustine, versus the investigator’s choice of Rituximab and Bendamustine or Rituximab, Gemcitabine, and Oxaliplatin [167].

Tazemetostat is now under evaluation for its feasibility and safety followed by SoC CAR T-cell therapy in patients previously treated with DLBCL, FL, and MCL in the trial, NCT05934838. The hypothesis is that this combination has the potential to significantly improve the ability of CART cells to recognize and kill lymphoma cells without a significant impact on safety. The participants will receive the oral tazemetostat before and after receiving their CAR T-cell therapy, for up to 12 months after CAR T-cell administration. Patients will be followed for up to 5 years.

Vorinostat is being evaluated under different combinations, in combination with Cladribine and Rituximab and with Cyclophosphamide, Etoposide, Prednisone, and Rituximab for elderly patients with R/R DLBCL, as already mentioned [180,181]. Study NCT03150329 is a phase 1 trial evaluating Pembrolizumab and Vorinostat in patients with R/R DLBCL, FCL, or HL. The rationale for the combination is that immunotherapy may help the body’s immune system attack the cancer and may interfere with the ability of cancer cells to grow and spread. Vorinostat may stop the growth of cancer cells by blocking some of the enzymes needed for cell growth. A study is also evaluating the toxicity and best dose of vorinostat when given together with pembrolizumab. NCT00972478 is evaluating Vorinostat, Rituximab, and combination chemotherapy in patients with newly diagnosed stage II, III, or IV DLBCL.

Among the 72 evaluable patients, with a median follow-up of three years, the estimated PFS at two years was 73%, while the OS was 86%. However, despite these outcomes, the regimen did not achieve the predefined efficacy improvement and was linked to high rates of febrile neutropenia (38%) and sepsis (19%). Given these limitations, it cannot be recommended for routine clinical use [212].

Tucidinostat is now being studied in trial NCT06779435 a prospective, observational, multicenter, cohort study of newly diagnosed DLBCL patients, trying to evaluate the clinical efficacy and safety of tucidinostat in a real-world setting. This trial was previously mentioned in this review.

NCT05272384 is a phase I clinical trial which is designed to evaluate the safety, side effects, and optimal dosing of nivolumab in combination with ASTX727 for the treatment of R/R B-cell lymphoma. Nivolumab, a monoclonal antibody used in immunotherapy, may enhance the immune system’s ability to recognize and attack cancer cells, potentially inhibiting tumor growth and spread. ASTX727 is a combination therapy consisting of Decitabine and Cedazuridine. Cedazuridine, a cytidine deaminase inhibitor, helps prevent the degradation of decitabine, thereby increasing its bioavailability and enhancing its therapeutic effect.

NCT05385263 is a phase 2 study evaluating the addition of Nivolumab to standard of care Anti-CD-19 CAR-T-cells in patients with stable/progressive DLBCL at lymphodepletion.

NCT03305445 is a multi-center, open-label trial that will enroll a single cohort of patients with R/R DLBCL who are ineligible for ASCT due to either resistance to salvage chemotherapy or the inability to tolerate high-dose myeloablative chemotherapy. All the participants will receive dual checkpoint blockade therapy, using doses established in phase III trials for ipilimumab and nivolumab. These agents will be administered at three-week intervals, with two doses given prior to and two doses following "immune transplant". During immune transplanting, T-cells within whole peripheral blood mononuclear cells (PBMCs) will be cryopreserved and later re-infused—a process known as adoptive T-cell transfer (ATCT). This will occur after a lymphodepleting chemotherapy regimen, following the current protocols for adoptive T-cell therapies.

NCT05507541 is an active recruiting phase 2 study with Safety Run-in of PD-1 Inhibitor and IgG4 SIRPα-Fc Fusion Protein (TTI-622) in R/R DLBCL. The primary objectives are to determine the toxicities of Maplirpacept (TTI-622) combined with pembrolizumab to identify the recommended phase 2 dose (RP2D) of TTI-622, combined with pembrolizumab and to estimate the preliminary efficacy of pembrolizumab in combination with TTI-622 as the measured ORR. The secondary objective is to estimate the efficacy of pembrolizumab in combination with TTI-622 as measured DOR, PFS, and OS.

NCT03995147 proposed to research untreated non-germinal center diffuse large B-cell lymphoma and what causes the disease and the way patients respond to pembrolizumab combined with R-CHOP chemotherapy.

NCT03630159 is a study of Tisagenlecleucel in combination with Pembrolizumab in r/r diffuse large B-cell lymphoma patients (PORTIA Trial). The combination of Tisagenlecleucel and Pembrolizumab was found to be feasible, with a manageable safety profile and no dose-limiting toxicities observed. Early signs of efficacy were noted when pembrolizumab was administered one day prior to Tisagenlecleucel; however, due to the small patient sample size and short follow-up period, definitive conclusions cannot yet be drawn. While adding pembrolizumab did not enhance the cellular expansion of Tisagenlecleucel, it was associated with a delayed peak expansion when administered the day before Tisagenlecleucel [213].

NCT03259529 is evaluating the safety and efficacy of a combination of Bendamustine, Gemcitabine, Rituximab, and Nivolumab (BeGeRN) in patients with R/R DLBCL (BeGeRN). The study is completed but the data are not published yet.

NCT03244176 is evaluating the feasibility of adding induction and maintenance Avelumab to the standard combination of R-CHOP in patients with stage II, III, and IV DLBCL. The primary endpoint is immune-related toxicity which requires the discontinuation of Avelumab. Secondary endpoints are response rates, failure-free survival, OS, and overall toxicity of the treatment.

NCT02596971 was a study of Atezolizumab in combination with either Obinutuzumab plus Bendamustine or Obinutuzumab plus CHOP in patients with FL or Rituximab + CHOP in patients with DLBCL. Data are published for FL, but not for DLBCL [211].

NCT03610061, the RaDD Study, is evaluating the safety profile of radiotherapy and durvalumab in R/R DLBCL and FL patients.

Tafasitamab is an active molecule in clinical trials at this time, with multiple clinical trials evaluating the efficacy in different combinations. NCT04978584 is a phase 2 trial that is studying the effect of rituximab, lenalidomide, acalabrutinib, and tafasitamab alone and in combination with chemotherapy for treating patients with newly diagnosed DLBCL, called the Smart Stop trial.

NCT05626322 is evaluating the effects of Maplirpacept (PF-07901801), Tafasitamab, and Lenalidomide when given together in R/R DLBCL.

NCT06299553, the PRO-MIND trial is an observational study now evaluating the effectiveness of Tafasitamab in combination with Lenalidomide followed by Tafasitamab monotherapy R/R DLBCL in patients who are not eligible for transplant in Italy.

NCT02763319 is a phase 2 and 3 trial that has been proposed to compare the safety and efficacy of Tafasitamab with BEN versus Rituximab with BEN in adult patients with R/R DLBCL who are not eligible for high-dose chemotherapy and ASCT. The trial is completed but no results have been posted yet. 

Another study, NCT05883709, which is not yet recruiting is evaluating Tafasitamab in combination with Lenalidomide in patients with R/R DLBCL in a real-world setting.

NCT06521255 is still recruiting for a phase 3 trial of Tafasitamab and Lenalidomide in combination with Gemcitabine and Oxaliplatin versus Rituximab in combination with Gemcitabine and Oxaliplatin in patients with R/R DLBCL.

NCT04974216 is a phase 2 study that will evaluate the efficacy of Tafasitamab and Lenalidomide associated with Rituximab in elderly patients with frontline DLBCL as assessed by the ORR after three cycles of treatment according to the Lugano Response Criteria.

RealMIND, NCT04981795, is an observational study on the safety and efficacy of Tafasitamab in combination with Lenalidomide in patients with R/R DLBCL.

Another study, NCT05552937, is evaluating the safety and efficacy of Tafasitamab combined with Lenalidomide in patients with R/R DLBCL.

CAR-T therapies are being extensively investigated in numerous clinical trials at this moment, with new drugs being investigated. We will mention the trials that are combining CAR-T with other therapies. NCT04889716 is an ongoing trial that is evaluating the safety and efficiency of the experimental drugs, bispecific antibodies, mosunetuzumab or obinutuzumab, and glofitamab given after CAR-T. The study is including patients who have already received a CAR-T-cell infusion. 

## 6. Conclusions

The dysregulation of epigenetics in lymphoma tumor cells and the surrounding tumor microenvironment (TME) has been described. Yet, there is still limited knowledge about the specific epigenetic modifications that link these two entities. The reciprocal interaction between tumor cells and TME components activates signaling cascades that lead to distinct changes in gene expression patterns driven by epigenetic mechanisms. Recent advancements in molecular pathology have significantly progressed lymphoma research, particularly in addressing the challenges associated with delayed early-stage diagnoses that hinder timely and effective treatment, often resulting in poor outcomes. Emerging evidence indicates that many epigenetic alterations in B-cell lymphomas occur early and are both inheritable and reversible. This presents potential therapeutic opportunities by targeting and reversing these mutations. While the role of epigenetic modifications has primarily focused on tumor cells in diffuse large B-cell lymphoma and other types, there is compelling evidence that the TME also plays a crucial role in promoting the proliferation, migration, and survival of lymphoma cells. Consequently, an increasing number of studies are exploring the epigenetic changes. All these changes occur in the TME and make a contribution to the immunosuppressive environment that supports tumor growth. To effectively combat lymphomas, a deeper understanding of the interplay between lymphoma tumor cells, the TME, and the involved epigenetic mechanisms is essential. The relationships between different cell types and the changes in gene activity related to the disease are complicated. This complexity poses a big challenge in everyday medical practice. However, a better understanding of how these cells interact could lead to new developments in lymphoma research and improve our knowledge of the disease. Epigenetic alterations may be used as biomarkers for lymphomas, allowing for a better patient stratification based on the expression of these epigenetic signatures. The use of epigenetic drugs may also provide a promising therapeutic option, addressing not only the elimination of lymphoma tumor cells but also counteracting the heterogeneous effects of the TME components. This approach may disrupt the interaction between cancer cells and immune cells, potentially slowing tumor growth and reducing the treatment resistance. It may also alter the immunosuppressive effects of the tumor microenvironment. Advancing research into epigenetic therapies is essential as it offers new avenues for diagnosing, treating, and predicting outcomes in lymphomas. As the clinical data on various therapies continue to emerge, determining the optimal treatment sequencing, identifying the most effective combination strategies, and defining the patient populations that would derive the greatest benefit remain key areas for further investigation.

## Figures and Tables

**Table 1:** Epigenetic regulators and their role in DLBCL and effect on TME cells.

Epigenetic Regulators	Incidence in DLBCL	Affected Genes	TME Modifications
cytidine deaminase CDA	40%	*BCL6*	Growth inhibition and apoptosis in DLBCL cells [28,29]
CREBBP	15%	*CD40*, *CD74*, *CIITA*, *IFN*, *MHC II*, *PD-L1*	Decrease in CD4 cells [62,83]
EP300	5%	*CDC25B*, *CDKN1A*, *E2F1*, *PDNA*	Increase in M2 Macrophages [84]
*EZH2*	25%	*CIITA*, *NLRC5*, *Th1-type chemokines*	Decrease in T_FH_ cells [85]
KMT2C	5%	*AP-1*, *ETS/PU.1*, *GPX8*, *GSTT1*, *GSTA4*, *IL1*, *IRF*, *RUNX*	Increase in CD8 T-cells and effector of immune cells [48,86]
KMT2D	30%	*CD40*, *IL10*, *IL6*	Increase in exhausted CD8^+^ T-cells and increase in effector immune cells [87]
TET2	10%	*CD40*, *IL10-IL6*, *MHC II*	Increase in CD^8^ T-cells and NK cells [88,89]

**Table 2:** Clinical trials targeting epigenetic modifications in DLBCL.

Clinical Trial	Mechanism	Phase	Disease	Results
NCT01120834 Azacitidine or Decitabine + Vorinostat [151]	DNMTi in combination with HDACi	I/II	R/R DLBCL	ORR 6.7% 3 month OS 77%
NCT02846935 Decitabine + tetrahydrouridine (THU) [154]	DNMTi in combination with CDA inhibitors	Pilot clinical trial	Aggressive B-cell and T-cell lymphomas	ORR > 30%
NCT02343536 Azacitidine + R-CHOP [155]	DNMTi in combination with R-CHOP	I	Newly diagnosed DLBCL, Grade 3B FL, transformed lymphoma	CRR 88.1%
NCT04799275 Azacitidine + R-miniCHOP [156]	DNMTi in combination with R-CHOP	II/III	Newly diagnosed DLBCL and associated aggressive lymphoma	1-year PFS 69% a projected 2-year OS of 71%
NCT03579082 Decitabine + R-DHAP [157]	DNMTi in combination with R-DHAP	IV	R/R DLBCL	ORR 40% vs. 33% PFS 7m vs. 5m OS 17m vs. 9m
NCT03494296 Low-dose Decitabine + Cyclophosphamide/Vindesine/Bonisone (COP)	DNMTi in combination with COP	Observational	R/R DLBCL	
NCT05816746 Decitabine + anti-PD-1	DNMTi in combination ICI	II	R/R DLBCL	
NCT06683885 Obutinib or Decitabine with Rituximab, Cyclophosphamide, and Prednisone	DNMTi in combination with chemotherapy	I/II	Newly diagnosed DLBCL	
NCT01622439 Valproate + R-CHOP [170]	HDACi Joint R-CHOP	I	DLBCL	2-year PFS: 84.7% 2-year OS: 96.8%
NCT00764517 Vorinostat + Cladribine + Rituximab [210]	HDACi in combination with CD20 monoclonal antibody-targeted drug	I/II	Relapsed NHL	PFS: 5.95 months OS: 0.95 months
NCT00667615 Vorinostat + Cyclophosphamide, Etoposide, Prednisone and Rituximab [171]	HDACi in combination with CD20 monoclonal antibody and chemotherapy	I/II	Relapsed DLBCL	ORR 55% mPFS 10mo
NCT03150329 Vorinostat + Pembrolizumab	HDACi + ICI	I	R/R DLBCL, FL, HL	
NCT00972478 Vorinostat + Rituximab + chemotherapy [209]	HDACi + chemotherapy	II	Newly diagnosed DLBCL	PFS 73% OS 86% High Toxicity
NCT02753647 Tucidinostat + R-CHOP [173]	HDACi Joint R-CHO	II	Newly diagnosed DLBCL	ORR: 94% CRR: 86% 2-year PFS: 68%, OS: 83%
NCT06779435 Tucidinostat in a real-world setting	HDACi	Observational	Newly diagnosed DLBCL	
NCT02282358 Mocetinostat	HDACi	II	R/RDLBCL or FL patients carrying CREBBP or EP300 mutations	1-year ORR: 14% PFS: 4.6 months, EFS: 4.6 months
NCT01897571 Tazemetostat + Prednisolon [164]	*EZH2* inhibitor	I	R/R B-NHLs and advanced solid tumors	ORR, including CRR) observed in 8 of 21 patients (38%) with B-NHLs
NCT03009344 Tazemetostat [162]	*EZH2* inhibitor	I	R/R B-NHLs	
NCT02220842 Tazemetostat + Atezolizumab [166]	*EZH2* inhibitor combined with monoclonal anti-PD-L1 antibody	Ib	R/R DLBCL	Median PFS: 2 months, median OS: 13 months
NCT02889523 Tazemetostat	*EZH2* inhibitor in treated patients with R-CHOP	Ib	Newly diagnosed DLBCL	
NCT05934838 Tazemetostat + CAR-T	*EZH2* inhibitor + CAR-T	I	Previously DLBCL, FL, and MCL.	
NCT0484287 Valemetostat	*EZH2* inhibitor	II	R/R aggressive B-cell lymphomas, transformed indolent lymphomas, FL, MCL, MZL, HL	
NCT04104776 CPI-0209	*EZH2* inhibitor	I/II	Advanced solid tumors R/R DLBCL	
NCT04390737 HH2853	*EZH2* inhibitor	I	Advanced solid tumors R/R DLBCL, FL	
NCT03460977 PF-06821497	*EZH2* inhibitor	I	DLBCL (single agent)	
NCT05272384 Nivolumab + ASTX727 (Decitabine and Cedazuridine)	ICI + DNMTi	I	RR B-cell lymphoma	
NCT05385263 Nivolumab + CAR-T	ICI + CAR-T	II	DLBCL treated with CAR-T targeting CD19	
NCT03305445 Nivolumab + Ipilimumab	ICI combination	I	R/R DLBCL ineligible for ASCT	
NCT05507541 TTI-662 + Pembrolizumab	ICI	II	R/R DLBCL	
NCT03995147 Pembrolizumab + R-CHOP	ICI + chemotherapy	II	Newly diagnosed B-cell lymphomas	
NCT03259529 Bendamustine + Gemcitabine + Rituximab + Nivolumab (BerGeN)	ICI + chemotherapy	I/II	R/R DLBCL	
NCT03244176 Maintenance Avelumab Plus R-CHOP	ICI + chemotherapy	Early I	DLBCL	
NCT02596971 Atezolizumab + R-CHOP [211]	ICI + chemotherapy	I/II	FL DLBCL	
NCT03610061 Durvalumab + Radiotherapy	ICI + radiotherapy	I	R/R DLBCL and FL	
NCT04978584 Tafasitamab, rituximab, lenalidomide, acalabrutinib	Sigle arm or combinations	II	Newly diagnosed DLBCL	
NCT05626322 Tafasitamab Maplirpacept (PF-07901801), and Lenalidomide	CD19 combination	I/II	R/R DLBCL	
NCT06299553 Tafasitamab + Lenalidomide, followed by Tafasitamab	CD19 + immunomodulatory	Observational	R/R DLBCL	
NCT02763319 Tafasitamab + BEN vs. R-BEN		II/III	R/R DLBCL	
NCT05883709 Tafasitamab+ Lenalidomide	CD19 + immunomodulatory	Observational	R/R DLBCL	
NCT06521255 Tafasitamab and Lenalidomide + GEMOX	CD19 + immunomodulatory + Chemotherapy	III	R/R DLBCL	
NCT04974216 Tafasitamab and Lenalidomide + Rituximab	CD19 + immunomodulatory + Chemotherapy	II	Newly diagnosed DLBCL in elderly	
NCT04981795 Tafasitamab and Lenalidomide	CD19 + immunomodulatory	Observational	R/R DLBCL	
NCT05552937 Tafasitamab and Lenalidomide	CD19 + immunomodulatory	II	R/R DLBCL	
NCT04889716 CAR-T Followed by Bispecific Antibodies		II	R/R DLBCL	

## Data Availability

No new data were created or analyzed in this study.

## Abbreviations

The following abbreviations are used in this manuscript:

ABC	Activated B-cell
AML	Acute myeloid leukemia
APRIL	Proliferation-inducing TNF ligand
BL	Burkitt lymphoma
B-NHL	B-cell non-Hodgkin lymphoma
CAF	Cancer-associated fibroblast
CHOP	Cyclophosphamide, Doxorubicin, Vincristine, Prednisone
CLL	Chronic lymphocytic leukemia
CRR	Complete response rate
DC	Dendritic cells
DLBCL	Diffuse large B-cell lymphoma
DNA	Deoxyribonucleic acid
DNMTs	DNA methyltransferases
DP-LME	Depleted lymphoma microenvironment
EBV	Epstein–Barr virus
ECM	Extracellular matrix
FL	Follicular lymphoma
GCB	Center B-cell subtype
GC-LME	Germinal center-like lymphoma microenvironment
HAT	Histone acetyltransferase
HDAC	Histone deacetylase
IL-10	Interleukin-10
LME	Lymphoma microenvironment
LSD1	Lysine-specific demethylase 1
MCL	Mantle cell lymphoma
MDSC	Myeloid-derived suppressor cell
MSD	Myelodysplastic syndrome
NGS	Next-generation sequencing
NK	Natural killer
NHLs	Non-Hodgkin lymphomas
ORR	Overall response rate
OS	Overall survival
PD-1	Programmed cell death protein 1
PD-L1	Programmed death-ligand 1
PFS	Progression-free survival
R-CHOP	Rituximab, Cyclophosphamide, Doxorubicin, Vincristine, Prednisone
RNA	Ribonucleic acid
RR-DLBCL	Refractory diffuse large B-cell lymphoma
TAM	Tumor-associated macrophage
TAN	Tumor-associated neutrophil
TILs	Tumor-infiltrating lymphocytes
Th	T helper
TME	Tumor microenvironment
TLA	Three letter acronym
Treg	T regulatory
